# Foundation model cascades enable zero-shot microscopy image analysis for cell therapy manufacturing

**DOI:** 10.1016/j.jcyt.2026.102078

**Published:** 2026-02-03

**Authors:** Rui Qi Chen, Yeonju Lee, Benjamin Joffe, Caroline E. Serafini, Paloma Casteleiro Costa, Bryan Wang, Bharat Kanwar, Stephen Balakirsky, Aaron D. Silva Trenkle, Linda E. Kippner, Isaac LeCompte, Ye Li, Christine E. Brown, Gabriel A. Kwong, Francisco E. Robles, Krishnendu Roy, Jing Li

**Affiliations:** 1 H. Milton Stewart School of Industrial and Systems Engineering, Georgia Institute of Technology, Atlanta, Georgia, USA; 2 Georgia Tech Research Institute, Georgia Institute of Technology, Atlanta, Georgia, USA; 3 Wallace H. Coulter Department of Biomedical Engineering, Georgia Tech and Emory University, Atlanta, Georgia, USA; 4 School of Engineering, Vanderbilt University, Nashville, Tennessee, USA; 5 The Marcus Center of Excellence for Biomanufacturing, Georgia Institute of Technology, Atlanta, Georgia, USA; 6 The Parker H. Petit Institute for Bioengineering and Bioscience, Georgia Institute of Technology, Atlanta, Georgia, USA; 7 Department of Hematology & Hematopoietic Cell Transplantation (T Cell Therapeutics Research Laboratories), City of Hope Beckman Research Institute and Medical Center, Duarte, California, USA

**Keywords:** anomaly detection, cell counting, cell therapy manufacturing, cell viability estimation, foundation models, large language models, microscopy image analysis

## Abstract

**Background aims:**

The scalable manufacturing of cell therapies creates a significant need for robust process analytical technologies, where automated analysis of noninvasive microscopy images offers a powerful method for monitoring critical quality attributes. However, conventional machine-learning models are often bottlenecked by extensive data labeling and poor generalizability across different batch effects. To overcome these limitations, we introduce a foundational model cascade for the zero-shot analysis of microscopy images.

**Methods:**

In the first stage, a multimodal large language model (LLM) detects anomalies, and anomalous images immediately trigger an alert. Otherwise, the segment anything model performs exhaustive instance segmentation, and the detected objects are classified by the LLM to estimate cell counts and viability.

**Results:**

This unified, zero-shot approach delivers robust anomaly detection together with quantitative measures of cell count and health, without any task-specific fine-tuning.

**Conclusions:**

By combining pre-trained foundation models in a complementary cascade, our method provides a generalizable solution for real-time process monitoring and feedback control, paving the way for more scalable and automated cell therapy manufacturing.

## Introduction

Cell therapy harnesses living cells as therapeutic agents to treat cancer, cardiovascular diseases, autoimmune diseases and neurologic disorders [1–4]. These treatments offer transformative benefits, including targeted disease eradication, long-lasting efficacy, and the potential to be personalized to each individual patient [5–7]. The field is rapidly advancing, with a growing number of therapies approved by the US Food and Drug Administration (FDA) and European Medicines Agency, including chimeric antigen receptor (CAR) T cells to treat various blood cancers and mesenchymal stromal cells to treat graft-versus-host disease [8–10].

Despite these successes, the greatest limitation of cell therapies remains patient access, largely due to the complex and costly manufacturing processes that struggle to consistently deliver high-quality cell products at scale [11]. Variability in patient- or donor-derived cell sources, combined with limited real-time quality monitoring, poses major challenges to maintaining consistent yield, safety and efficacy [12]. From a regulatory standpoint, both the FDA and European Medicines Agency emphasize the need to monitor Critical Quality Attributes (CQAs) throughout the manufacturing process, including viable cell numbers, cell concentration, cell health and the detection of anomalies or impurities. These requirements are intended to ensure product safety, reproducibility and compliance with established release criteria, including minimum viability thresholds, defined cell doses and limits on contamination or unexpected cell types. To meet these imperatives, there is a pressing need for streamlined and automated manufacturing systems that incorporate quality-by-design principles and active process control. Such systems would support reproducible, high-quality cell production while minimizing failure rates, reducing costs and improving patient access. One promising approach involves the development of automated manufacturing workflows that integrate real-time sensing technologies to monitor critical process parameters and product attributes. These systems aim to enable dynamic control and decision-making throughout production, improve consistency, reduce manual intervention and ensure the reliable generation of high-quality cell therapy products [13].

To meet this need for real-time process monitoring, label-free imaging techniques offer a particularly powerful solution. Among these, quantitative oblique back-illumination microscopy (qOBM) is a scalable method that can reconstruct images within minutes of capture, making it well-suited for functional imaging of cells directly within bioreactors [14–17]. This technology enables the continuous, non-invasive monitoring of cell quality throughout the manufacturing process, providing a wealth of data that can be used to optimize the therapeutic product [18]. However, translating this stream of raw image data into actionable insights for automated manufacturing requires sophisticated analytical tools. Although traditional machine learning models can be trained for this purpose, they often struggle with the inherent variability between manufacturing runs and require extensive, costly and time-consuming efforts to label data and retrain models for each new task or condition [19]. This work addresses the gap by introducing a zero-shot foundation model cascade capable of analyzing qOBM images without the need for task-specific training, thereby enabling a more agile and scalable approach to automated process control.

Our primary interest in analyzing microscopy images is to quantify CQAs that are both scientifically and regulatorily critical: cell population density (counting), cell viability (live versus dead) and the detection of anomalies such as imaging artifacts or foreign contaminants (e.g., yeast or bacteria) [20]. To accomplish these tasks simultaneously, we developed a foundation model cascade that operates in a zero-shot capacity. In the first stage, a multimodal large language model (LLM) performs anomaly detection, immediately alerting the user if anomalies are detected, thereby preventing compromised data from influencing downstream analysis. For non-anomalous images, the segment anything model (SAM) performs exhaustive, class-agnostic instance segmentation to identify individual cells [21]. The LLM then evaluates each segmented object to classify its viability and generate cell counts. This unified and interpretable framework simultaneously provides robust anomaly detection, accurate cell counting and viability estimation, directly supporting regulatory requirements for reproducibility, batch release criteria and product safety.

**Fig. 1.**
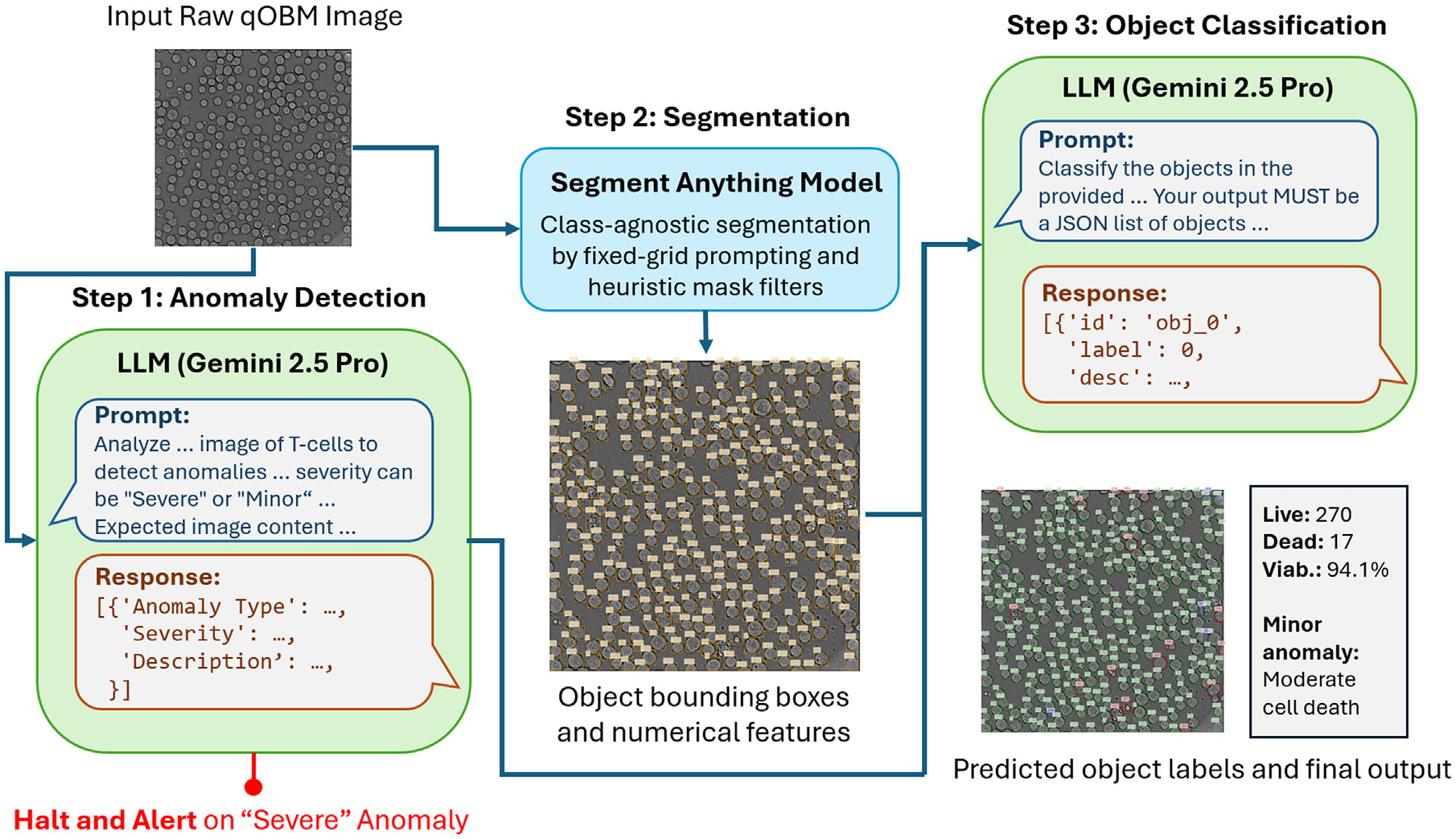
The proposed cascade. (1) An input qOBM image is first evaluated by an LLM guided by a prompt to screen for process anomalies. If a severe anomaly is detected, the analysis is halted and an alert is generated. (2) If the image is normal, it is processed by SAM to perform class-agnostic instance segmentation. (3) The resulting object bounding boxes and extracted numerical features are passed back to the LLM to classify each object. The final outputs include a list of detected anomalies, a list of detected objects and their class labels, aggregated cell counts and culture viability.

In this work, we evaluate the performance and robustness of our proposed cascade in a diverse set of T-cell qOBM images that feature various imaging conditions and types of anomalies, demonstrating its effectiveness without any parameter tuning. In addition, we conducted a systematic ablation study to quantify the impact of each component on our prompting architecture. We compare multiple prompting strategies, beginning with a simplistic baseline and progressively incorporating more advanced techniques: providing explicit domain knowledge, adding SAM for instance segmentation, extracting numerical features for the LLM and mandating rationale generation. The overview of the final cascade can be seen in Figure 1.

To summarize, this work makes the following contributions:

- We propose a novel, zero-shot architecture that cascades foundation models to perform interpretable and generalizable analysis of cell culture microscopy images including anomaly detection, cell counting and viability estimation.
- We provide a systematic ablation study that quantifies the performance gains from different prompt components, offering insights into the effective design of LLM-based analysis frameworks.
- We demonstrate the robustness of the cascade across diverse imaging conditions, anomaly types, cell densities and viability levels. Validation on three external datasets containing T cells and CAR T cells from more than 40 donors yielded <0.1 mean absolute error (MAE) on viability prediction, reinforcing its utility for real-world manufacturing scenarios.

## Related Works

### Cell microscopy image analysis

A typical cell image analysis pipeline involves segmenting cells from the background, extracting features, followed by unsupervised clustering and dimensionality reduction to identify patterns, or classifying cells into predefined categories [22,23]. Early image analysis methods relied on classical image processing combined with handcrafted texture and morphology descriptors. CellProfiler introduced a modular workflow of illumination correction, global/adaptive thresholding and watershed separation to segment cells [24]. QuPath extended this paradigm to whole-slide tissue images, adding interactive object classification and batch scripting [25]. Despite these developments, these approaches require extensive human input to tune and are sensitive to domain shifts, limiting their robustness for real-time, large-scale manufacturing environments where variability is inherent and manual intervention is costly.

The U-Net that won the Cell Tracking Challenge at the 2015 International Symposium on Biomedical Imaging showed that convolutional neural networks can learn rich features from a small number of annotated images, quickly becoming the de facto baseline for segmentation tasks [26]. Subsequent work also has adopted various deep learning architectures for different tasks and imaging modalities [27–30]. More recently, modern architectures have further advanced dense prediction tasks in medical imaging. For instance, INSPIRE introduces scale-aware pyramidal feature learning to address intra-scale information loss in hierarchical networks [31]. Generative artificial intelligence has been applied to microscopy for virtual staining, such as PhaseFIT [32], which transforms label-free organoid images into virtual immunofluorescent images. Counterfactual reasoning has been explored to reduce bias in image-based analysis by disentangling spurious multimodal correlations that can distort visual feature learning [33]. For anomaly detection, deep learning–based generative or feature embedding strategies have been applied [19,34]. However, these methods require task-specific fine-tuning and retraining, making them poorly suited for adapting to new tasks without additional labeled data.

More recent advances in cellular image analysis have explored leveraging the generalization ability of foundation models trained on large and diverse datasets. SAM [21], SAM 2 [35] and SAM 3 [36] are a series of powerful prompt-based instance segmentation foundation models. They are often fine-tuned or paired with supervised components to adapt to biomedical tasks [37–40]. In computational pathology, foundation models like Virchow2 [41] have been adapted to specialized tasks such as pancancer survival prediction [42]. Yet, these models typically need task-specific training data, operate as standalone components, require a human-in-the-loop to use or lack the ability to perform contextual, multi-step analysis without supervision.

### Multimodal foundation models for zero-shot image analysis

Vision language models (VLMs) such as contrastive language-image pre-training (CLIP) [43] and LLMs such as Gemini 2.5 Pro [44] are trained on vast datasets that align images with natural language, enabling cross-modal reasoning and open-ended visual understanding. These models align visual features with natural language, enabling semantic understanding, output interpretability and prompt-based flexibility that traditional vision-only systems lack. Given their generalizability, these models can perform zero-shot classification, captioning, anomaly detection and even reasoning-based analysis across a wide range of scientific domains.

To enhance the domain relevance of contrastive models such as CLIP, many studies have explored fine-tuning strategies, adapter-based techniques and contrastive reweighting methods [45]. Domain-adapted variants such as BiomedCLIP [46] and PLIP [47] are trained on biomedical image–text pairs, improving sensitivity to specialized semantics while retaining support for zero-shot querying. In digital pathology, foundation models leveraging large-scale clinical data have demonstrated significant utility. Prov-GigaPath combines vision-only models with vision-language alignment on pathology reports to enable zero-shot subtyping [48], whereas CHIEF establishes a general-purpose framework by integrating unsupervised tile-level pretraining with weakly supervised whole-slide learning guided by CLIP-encoded anatomical priors to generalize across diverse cancer evaluation tasks [49]. For parameter-efficient adaptation, methods like M^2^PT introduce learnable visual and textual prompts to VLMs, achieving strong zero-shot generalization while tuning less than 0.1% of model parameters [50]. Once fine-tuned, VLMs can be directly applied to biomedical tasks such as hematology image classification without further supervision [51].

In contrast, autoregressive models offer more expressive prompting capabilities, enabling strategies such as Chain-of-Thought reasoning [52], visual grounding via Set-of-Mark prompts [53], and in-context learning [54]. These techniques allow even general-purpose LLMs to perform effectively in domain-specific applications, ranging from answering medical questions and interpreting microscopy images [55–57] to recognizing visual degradations for video reconstruction [58]. Consequently, no prior work has fully realized the potential of combining multimodal prompting with instance-level segmentation to create a task-agnostic yet interpretable analysis framework for microscopy-based process monitoring.

## Materials and Methods

### Data acquisition

#### Development dataset

The images in this dataset were obtained from multiple T-cell expansion experiments in batch bioreactors (institutional research board protocol no. H17348). A subset of images from this dataset was used for the systematic prompt development described in the section “Systematic development of the prompting framework.” The images have dimensions of 2048 by 2048 pixels, with a single channel measuring the quantitative phase. For downstream analysis, the images were converted to grayscale by linearly mapping a fixed intensity range to 0–255 to enhance contrast; values outside this range were clipped.

A total of 116 images were collected for this dataset, spanning 12 different experimental procedures, two different donors, culture ages ranging from 5 days to 14 days, and two imaging conditions designed to capture larger batch effects. Of these, 98 images were taken from samples pipetted onto a slide and 18 from samples imaged in-line using a custom flow cell. The imaging setting introduced systematic differences: while the flow cell enables continuous, in-line monitoring of cultures, its images are typically noisier with a grainy appearance compared to slide-based imaging.

**Fig. 2.**
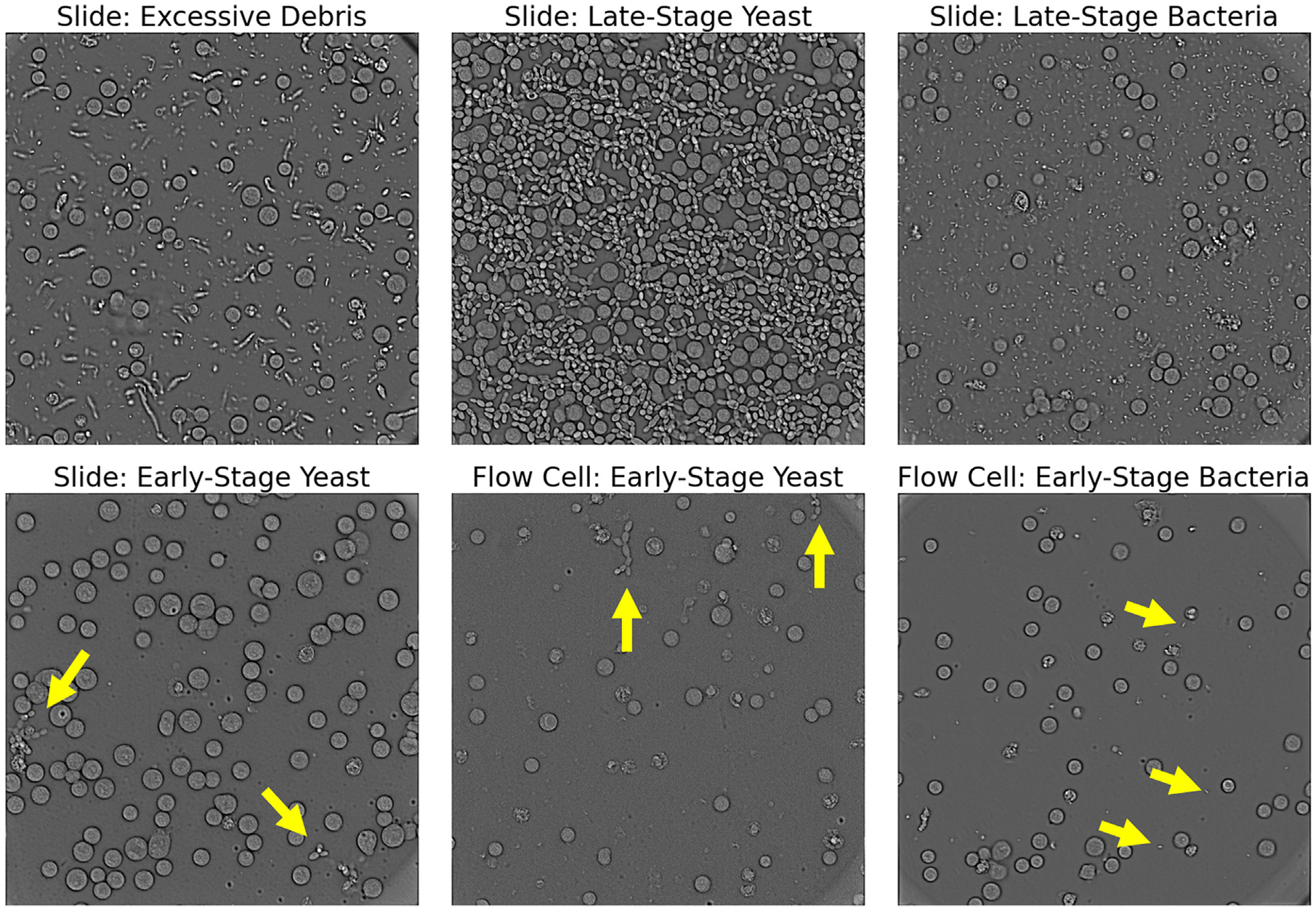
Examples of the severe anomalies present in the development dataset. Debris and late-stage contamination span the entire image, whereas early-stage yeast and bacterial contamination is more localized. The yellow arrows point to localized yeast and bacteria in early-stage contamination examples.

In this dataset, 22 images exhibited severe anomalies, which are defined in this work as deviations that can critically compromise the culture or prevent reliable analysis. The severe anomalies in the dataset were categorized into excessive debris (n = 3), early-stage yeast contamination (n = 7), late-stage yeast contamination (n = 2), early-stage bacterial contamination (n = 3), and late-stage bacterial contamination (n = 7). Examples of anomalous images can be seen in Figure 2. In this work, high cell death in images with low viability was defined as a minor anomaly, allowing the cascade to proceed with the viability analysis in the subsequent step. The detection of minor anomalies is designed to only be informative and would not halt the cascade.

For non-anomalous images, SAM was first used to place bounding boxes around candidate objects. A subset of the segmented masks were discarded if they met any of the following criteria: an area smaller than 38 *μ*m^2^ (2000 pixels), area greater than 1142 *μ*m^2^ (60000 pixels), predicted intersection over union (IoU) below 0.5, or stability score below 0.5. These thresholds were applied to exclude objects unlikely to represent T cells. Then, redundant detections were removed using non-maximum suppression with an IoU threshold of 0.2. The remaining objects were then manually classified as live cells, dead cells, or non-cellular objects, following the criteria of [19], to generate ground truth labels for evaluation. The cell density in these images ranged from 68 cells per image to 302 cells per image, and viability ranged from 0.28 to 0.97. In total, 8901 objects were labeled as live cells, 1693 as dead cells, and 931 as non-cellular.

#### External test datasets

To ensure the robustness of the segmentation and object classification steps, we evaluated the system on three external qOBM datasets that did not influence the design of the segmentation or object classification prompts. Ground truth labels for these datasets were obtained from flow cytometry or a fluorescent stain assay. Datasets A and B contained no anomalies, whereas all images in dataset C exhibited excessive debris. To ensure representation of this anomaly mode, a subset of images from dataset C was included in the development dataset; however, these samples did not influence the design of the object classification prompts used to evaluate dataset C. Further details regarding these datasets can be found in Serafini *et al*. [18].

##### Dataset A: isolated cell culture

This dataset comprises 44 samples of primary human T cells from three donors (institutional research board protocol no. H20288). Samples were taken at variable culture ages, ranging from day 0 to day 21. Each sample was imaged at 1–6 different fields of views, resulting in 207 distinct images. Unlike the development set, these samples underwent density gradient centrifugation and CD4^+^ (n = 99) and CD8^+^ (n = 108) positive isolation. Cell densities were significantly lower than the development set, with SAM detecting between 11 and 97 objects per image. Viability was measured via flow cytometry (Cytek Northern Lights) using aqua Live/Dead staining. The measured viability values ranged from 0.30 to 0.99.

##### Dataset B: CAR T cells

This dataset consists of 258 images from 82 samples of CAR T cells derived from a cohort of 43 clinical trial participants, providing significant biological diversity across a large donor pool. The images captured cells at two distinct physiological states: immediately following a fresh thaw (n = 128) and after 24 hours of recovery in media (n = 130). Cell densities were similar to the development set, with SAM detecting between 64 and 329 objects per image. Like Dataset A, the viability was measured via flow cytometry, ranging from 0.32 to 0.96.

##### Dataset C: per-cell stain

This dataset contains 19 images with per-cell live/dead labels. Samples were treated with 4′,6-diamidino-2-phenylindole, then pipetted onto a slide and imaged with qOBM and fluorescence. By overlaying the fluorescence and phase channels, individual cells were labeled as alive or dead based on nuclear staining. In total, 661 cells were labeled as alive, and 104 as dead. The cell density in these images ranged from 4 cells per image to 100 cells per image.

#### Dynamic quantitative qOBM

Using epi-illumination, qOBM facilitates label-free three-dimensional quantitative phase imaging. Although this technology is adept at imaging thin and transparent samples, such as *in vitro* cells, similar to traditional phase imaging techniques, it also possesses the ability to analyze complex scattering samples that are incompatible with conventional methods, including thick tissues [59] and cells within bioreactors [14,18]. The qOBM system is built on a standard bright-field microscope setup and can be miniaturized into a flexible, fiber-based system [60], a handheld device [61] or a compact system that fits inside an incubator (as in this work), for a wide range of uses. Four low-cost LEDs provide the illumination for qOBM, coupled to 1-mm multimode fibers that sequentially illuminate the sample in epi-mode. These fibers are arranged at 90° intervals around the microscope objective. The light from the light-emitting diodes, delivered through the fibers, penetrates the sample and undergoes multiple scattering events. This scattered light effectively creates a virtual light source within the sample, mimicking a transmission-based microscope. For thin samples, a scattering medium with well-defined properties, such as a polydimethylsiloxane device with titanium dioxide nanoparticles, can be utilized to generate the back illumination (as was done in this study). The reconstruction of a quantitative phase image is accomplished by deconvolving the raw intensity images with the system’s transfer function. Further details on the system hardware and the reconstruction algorithm are available in previous publications [16,15,60]. The resulting processed qOBM images yield quantitative phase data proportional to the optical path length of the sample (the product of its refractive index and thickness), with a sensitivity of <2 nm. A lateral resolution of approximately 0.63 *μm* is achieved when using a 60×/0.7 NA objective and illumination at a wavelength of 720 nm.

#### Zero-shot foundation model cascade

Our approach to zero-shot image analysis is centered on a cascaded architecture that integrates distinct foundation models. We selected Gemini 2.5 Pro as the LLM due to its advanced capabilities, including its long context window for processing extensive input, its high-fidelity image understanding, and its native ability to interpret spatial references like bounding box coordinates [44]. The temperature for output generation was set at 0.5 to promote analytical responses rather than creative ones. The core of our method lies in how we interact with this model. The main instructions were included in the system instructions, whereas data-specific input such as the image and the SAM-detected objects were included in the remaining prompt contents. The development of this prompting architecture, detailed below, was the result of systematic study designed to progressively improve performance, interpretability, and reliability. Our final architecture consists of three primary stages:

1. The analysis begins with the detection of anomalies in the microscopy image using Gemini 2.5 Pro [44]. The LLM is prompted to screen for any large-scale process anomalies, such as contamination or significant imaging artifacts that require immediate attention or could impede further analysis. If a severe anomaly is detected, the analysis is stopped and the user is alerted.
2. In the second stage, the image is processed using SAM. Given the generalizability of SAM, it is always seeded with a fixed, 64 × 64 uniform grid of points. In a zero-shot, class-agnostic manner, SAM generates a comprehensive set of masks for all potential objects within the image, ranging from individual cells to debris and other artifacts. Then, heuristic filters based on size and segmentation confidence scores are used to filter out obvious non-cellular objects. This step effectively decouples object localization from subsequent analytical tasks.
3. Finally, the bounding box coordinates and extracted numerical features for each detected object are passed back to the LLM for detailed classification. The resulting object-level classifications are then aggregated to derive CQAs for the manufacturing process. The total cell count, which correlates with cell concentration, is determined, and the culture’s overall viability is calculated as the proportion of live cells to the total cell population.

#### Systematic development of the prompting framework

The core of the method lies in the design of prompts that guide the LLM. Separate prompts were developed for anomaly detection and for cell counting/viability estimation, reflecting the distinct challenges of each task. Our strategy was to begin with a minimal baseline and add guidance only where necessary, striking a balance between leveraging the model’s existing subject-matter knowledge and compensating for its known limitations. For example, while LLMs can draw on broad biomedical knowledge to reason about cell health, they require explicit instructions to distinguish between severe and minor anomalies, as this distinction is subjective without the user’s specifications. Similarly, because LLMs are notoriously poor at counting, especially at the scale of hundreds of objects, we introduce SAM to provide object-level grounding, with the LLM focusing instead on classification. This design philosophy ensures that prompts remain concise and targeted, offering structure only where the model would otherwise fail, while leaving space for the LLM to apply its own domain knowledge effectively.

Therefore, the systematic design process emphasized complementing, rather than replacing, the LLM’s existing knowledge. Starting from a minimal baseline, new instructions were introduced only when the model lacked the contextual grounding needed to reliably apply its subject matter expertise. Each addition was evaluated with respect to accuracy, prompt complexity, and inference cost, ensuring that the guidance remained lightweight and purposeful. By building prompts in this targeted, stepwise manner, the framework supports consistent outputs while retaining efficiency, offering a procedure that can be readily adapted to other domains of image analysis.

#### Anomaly detection

The anomaly detection prompts were constructed incrementally, with each stage adding specific context to improve the LLM’s ability to interpret microscopy images accurately. Four prompt versions for anomaly detection, referred to as AD-1 through AD-4, were created to capture progressively richer guidance. The complete system instructions for each prompt version can be found in Section A of the Supplementary Materials. All outputs were constrained to a fixed structured-output JavaScript Object Notation (JSON) schema, ensuring consistency and machine readability [62]. The output is parsed as a JSON list of anomalies, each with fields of “Anomaly Type,” “Severity” and “Description.”

##### AD-1: baseline detection

This initial zero-shot prompt tasks the instruction-tuned, reasoning-enabled LLM to identify anomalies and assign a severity of “severe” or “minor,” outputting results in a standardized JSON format. No further context about the expected image content is provided, representing the most minimal instruction set.

##### AD-2: expected content guidance

Building on the baseline, this prompt introduces information about normal image composition, indicating that live and dead T cells are the primary objects and that occasional non-cellular objects (specks or bubbles) are normal. It also specifies that widespread cell death (>20%) should be classified as a minor anomaly, unless it is accompanied by other irregularities.

##### AD-3: severity definitions

This version formalizes the distinction between minor and severe anomalies. Minor anomalies are defined as slight deviations with limited impact on analysis, whereas severe anomalies are those that critically compromise the culture or prevent reliable image interpretation. Examples include contamination, pervasive debris, and major image artifacts.

##### AD-4: detailed contextual guidance

The most comprehensive prompt adds explicit descriptions of normal and abnormal structures. The appearances of normal T cells, and non-cellular specks and bubbles are described. Potential severe anomalies are further detailed with specific examples, including bacterial, fungal, or mycoplasma contamination, cross-contaminating cells, refractile or crystalline debris, and focus or imaging artifacts that obstruct analysis.

#### Cell counting and viability estimation

The prompts for cell counting and viability were developed incrementally to provide the LLM with sufficient context to classify individual objects reliably while keeping outputs concise and grounded to reduce inference costs and prevent hallucination. Five prompt versions for counting and viability (CV), referred to as CV-1 through CV-4, were created to gradually introduce object-level grounding and additional information. The complete system instructions for each prompt version can be found in Section A of the Supplementary Materials. SAM is incorporated starting with CV-2 to provide explicit object detections that the LLM can reference using unique identifiers. The CV prompts did not explicitly include domain knowledge, as it led to worsened performance (see Section B for details).

##### CV-1: basic counts

The simplest zero-shot prompt instructs the model to count live and dead cells in the image and output the results as a JSON object. There is no guidance about cell morphology or distinguishing non-cellular objects, so the model is expected to rely exclusively on its existing domain knowledge. The total cell count is the sum of reported live and dead cells, and the predicted viability is the proportion of live cells to the total cell count.

##### CV-2: object-level grounding with SAM

With the introduction of SAM, the prompt provides a list of detected objects, each with a unique identifier and bounding box. The LLM now classifies each object as a “live cell,” “dead cell” or “non-cellular object,” producing a JSON map keyed by object IDs. To minimize output token use, the output is instructed to be a map of each object’s id The integer representation of the predicted class label. This structure also prevents drift in sequence-to-sequence tasks and ensures consistent correspondence between input objects and output classifications. This object-based classification improves both the reliability and interpretability of the outputs, because it enables the model to reason over localized visual evidence for each object, mitigating the risk of hallucinated or duplicate counts that can occur in image-wide estimation. For cell counting and viability prediction, non-cellular objects were excluded from the counts.

##### CV-3: feature-enriched object classification

This prompt supplements object-level information with numerical features. These features provide quantitative evidence that complements the model’s visual analysis. The included features are objectness score, area, solidity, eccentricity, standard deviation of intensity, gray-level co-occurrence matrix contrast and gray-level co-occurrence matrix homogeneity [63]. The objectness score is defined as the predicted IoU value from SAM, and the remaining features are computed with the SAM mask of each object and the grayscale phase image using functions available in NumPy and scikit-image [64,65]. By providing numerical features, the LLM can leverage both its visual and language capabilities to inform object classification. The mean and standard deviation of each feature are also provided to help contextualize individual values. The LLM uses these to refine classifications while maintaining a JSON map keyed by object IDs, ensuring minimal output token usage and improved robustness for large numbers of objects.

##### CV-4: explainable object classification

The final prompt extends CV-3 by requesting a brief textual rationale for each classification in addition to the object ID and label. The output is a JSON list of objects with id, label, and desc, providing interpretability for downstream review while preserving object-level grounding to prevent hallucination or misalignment.

## Results

### Experimental setup

The AD and CV prompts were evaluated on the 116 images in the development dataset. Normal images were used for both tasks, whereas anomalous images were included only in the anomaly detection evaluation.

Anomaly detection was formulated as a binary classification task, with severe anomalies as the positive class and minor or no anomalies as the negative class. Model performance was measured using accuracy, precision, recall, and F1 scores, with images predicted as anomalous (positive) if the model predicted at least one “Severe” anomaly. Cell-counting accuracy was assessed by calculating mean absolute percent error (MAPE) between predicted and ground truth cell counts (live cells + dead cells). The counts of live and dead cells allowed the calculation of the viability score, which was evaluated using MAE. Object-level classification performance was measured by classification accuracy and F1 scores for each class.

The images in the external test datasets were evaluated using SAM segmentation and CV prompts. For test datasets A and B, predicted cell viability was directly compared with ground-truth viability measurements obtained from flow cytometry. Results are reported on a per-image basis, as well as on a per-sample basis when multiple images were acquired from the same sample. Per-sample viability was calculated by aggregating cell counts across all images associated with that sample. In dataset C, the ground-truth labeled cells constituted a strict subset of the objects detected by SAM. To enable a meaningful comparison with the machine learning baseline in [18], only these labeled objects were provided to the LLM for classification, and the prompts were minimally modified to perform binary live/dead classification instead of three-class classification. Although some images in this dataset exhibited severe anomalies of “excessive debris,” they were nonetheless included in the evaluation. Ground-truth labels derived from 4′,6-diamidino-2-phenylindole staining were used to assess object-level classification performance.

To account for response variability, each prompt was run five times per image, with results reported as mean and standard deviation. Lastly, the cost of running each strategy was evaluated, based on the number of tokens used for each inference.

### Anomaly detection

The anomaly detection performance is shown in Table 1. The simplest baseline prompt, AD-1, had the highest recall, but at the cost of low precision, indicating that the model was eagerly assigning severe anomalies to many normal images. Describing the expected image content in AD-2 and defining the meanings of severe anomalies helped decrease the rate of false positive anomaly detection. Overall, AD-4 performed the best with the highest accuracy, precision, and F1 score, as well as high recall, suggesting that descriptions of potential anomalies can help direct the model’s focus to relevant observations. The evaluation showed a relatively low standard deviation, indicating limited variability in model responses.

**Fig. 3.**
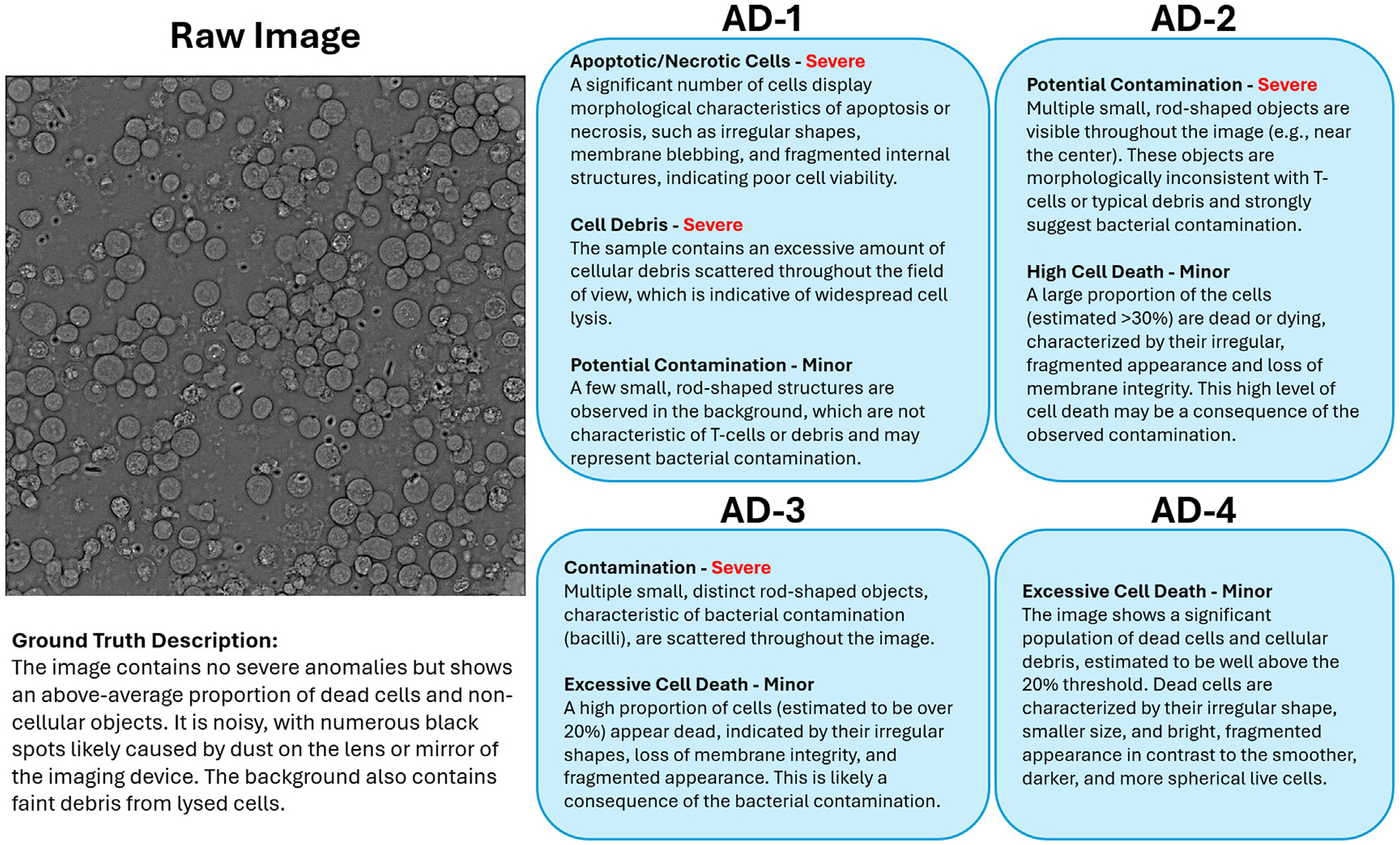
The impact of adding incrementally more guidance in the prompt for anomaly detection. The raw image and the observed description are on the left and the LLM-detected anomalies are on the right. Prompts with less guidance predicted more false positive anomalies than prompts with more guidance.

Figure 3 compares the anomaly detection output for a relatively challenging image as the prompt progresses from AD-1 to AD-4. With AD-1, the model output three anomalies, falsely detecting contamination and describing cell death and debris in overly severe terms, despite the image only showing reduced viability and lens dust. AD-2 added expectations about typical image content, which correctly revised excessive cell death as only a minor anomaly. However, it still misinterpreted the dust in the image as microbial contamination, indicating that without explicit descriptions of acceptable normal artifacts, the model’s existing knowledge could not accurately comprehend what it observed. In AD-3, the introduction of explicit severity criteria could not correct the misinterpretation of the dust. Finally, with AD-4, the addition of detailed rules and concrete descriptions of common non-cellular objects allowed the model to correctly identify the minor anomaly of excessive cell death above the 20% threshold, while dismissing normal dust and avoiding false contamination calls.

**Fig. 4.**
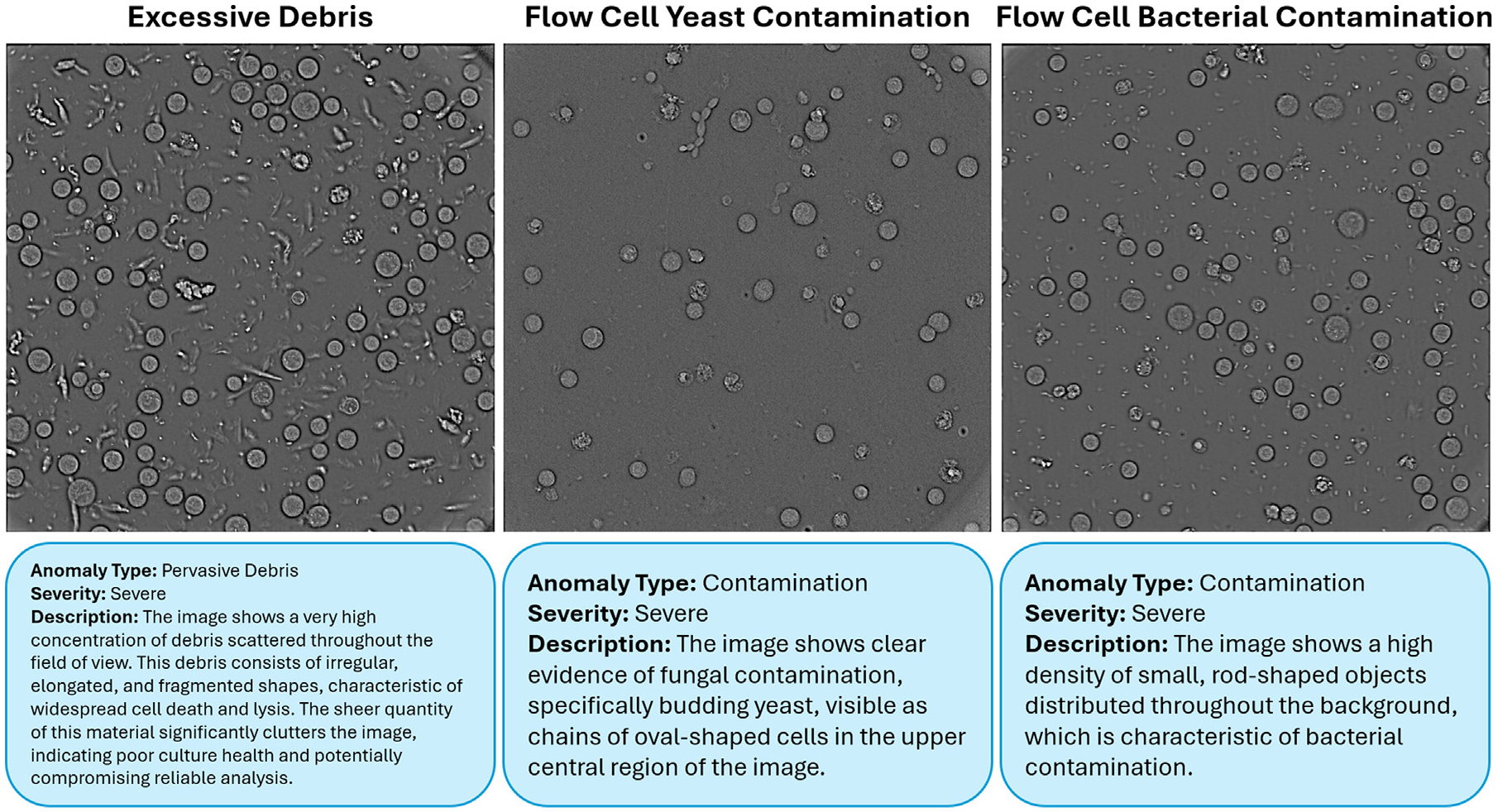
AD-4 correctly identifies and describes various process anomalies. Each column displays an input qOBM image alongside the anomaly detection portion of the output. From left to right: an exceptionally high concentration of bright, non-cellular debris; early stage, localized yeast contamination; and a population of late stage bacterial contamination.

Example capabilities of AD-4 are presented in Figure 4, showcasing its performance on three distinct categories of process anomalies. The model not only correctly identified the presence of an anomaly but also provided a detailed description of the visual characteristics it used to conclude the type of anomaly. For instance, it distinguished between a high concentration of elongated non-cellular debris, the initial emergence of oval-shaped structures indicative of early-stage yeast contamination, and a denser, rod-shaped population of bacteria. With this reasoning, the model not only identified the type of anomaly, but also specified its location when localized, making the output both auditable and actionable.

### Cell counting and viability estimation

The internal validation performance for cell counting and viability prediction is presented in Table 2. Although CV-1 did not use SAM for object-level classification, it achieved surprisingly competitive viability in MAE. However, without object-count grounding, it struggled with higher error rates for cell counts, hurting the interpretability of the viability prediction. The addition of SAM segmentation reduced cell counting errors, but at the cost of increased viability estimation errors. Only through the inclusion of numerical features was a reduction in both cell counting and viability errors achieved. With SAM and numerical features, the model could achieve a relative error of less than 10% in cell counting and an absolute error of less than 5% MAE in viability prediction in both flow cell and slide settings. In terms of prediction errors, the optimal prompting strategy appears to be dependent on the imaging setting. The additional task of outputting object classification rationales in CV-4 yielded the best performance in the flow cell setting, while CV-3 is the best for the slide setting. The flow cell setting showed higher variability in model responses, as these images typically contained fewer cells and were noisier, which limited the model’s ability to capture sufficient context and led to less consistent interpretations across repetitions. In sum, both CV-3 and CV-4 represent substantial improvements over CV-2 and are acceptable for accurate cell counting and viability prediction.

**Fig. 5.**
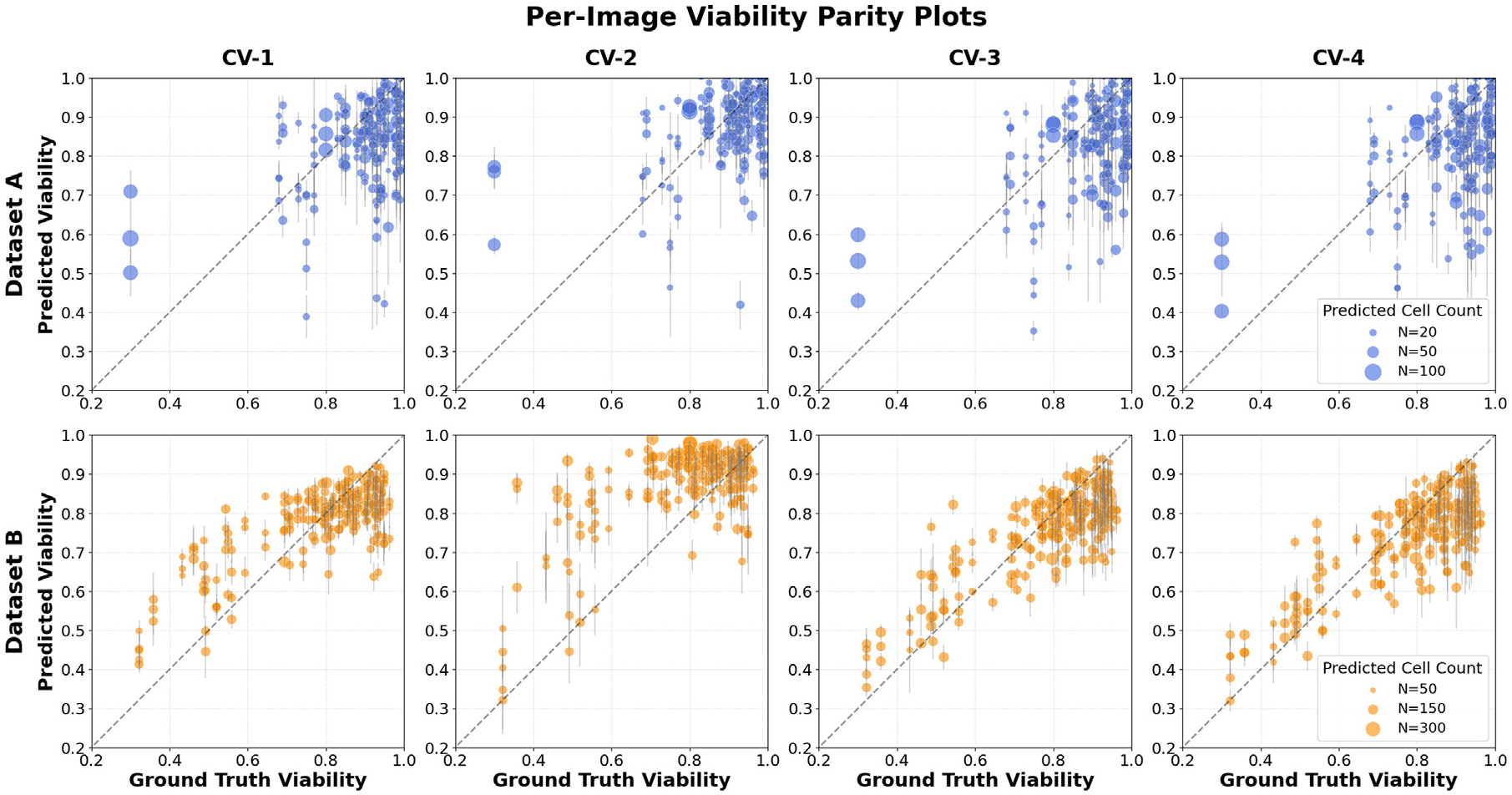
Zero-shot generalization per-sample viability parity plots for external datasets A and B. Data points and error bars show the mean and standard deviation across five repetitions, respectively. The size of each data point represents the total number of cells detected by the cascade. The top row shows dataset A, and the bottom row shows dataset B. The columns from left to right show CV-1, CV-2, CV-3 and CV-4. Images with fewer detected cells tended to show lower viability and higher errors.

The viability prediction results for test datasets A and B are shown in Table 3, and parity plots are shown in Figure 5. For both datasets, the prediction errors are greater than those observed on the development dataset. This increase can be partially attributed to sampling variability, as evidenced by improved accuracy when predictions are aggregated across multiple images. Notably, the numerical features used in CV-3 and CV-4 resulted in reduced prediction accuracy for Dataset A.

As a point of reference, the baseline machine learning approach reported in [18] achieved MAE values of 0.0389 and 0.0227 on datasets A and B, respectively. However, these results are not directly comparable, since [18] employed a different cell segmentation method, performed binary classification, and restricted analysis to samples containing more than 100 cells.

### Object classification

**Fig. 6.**
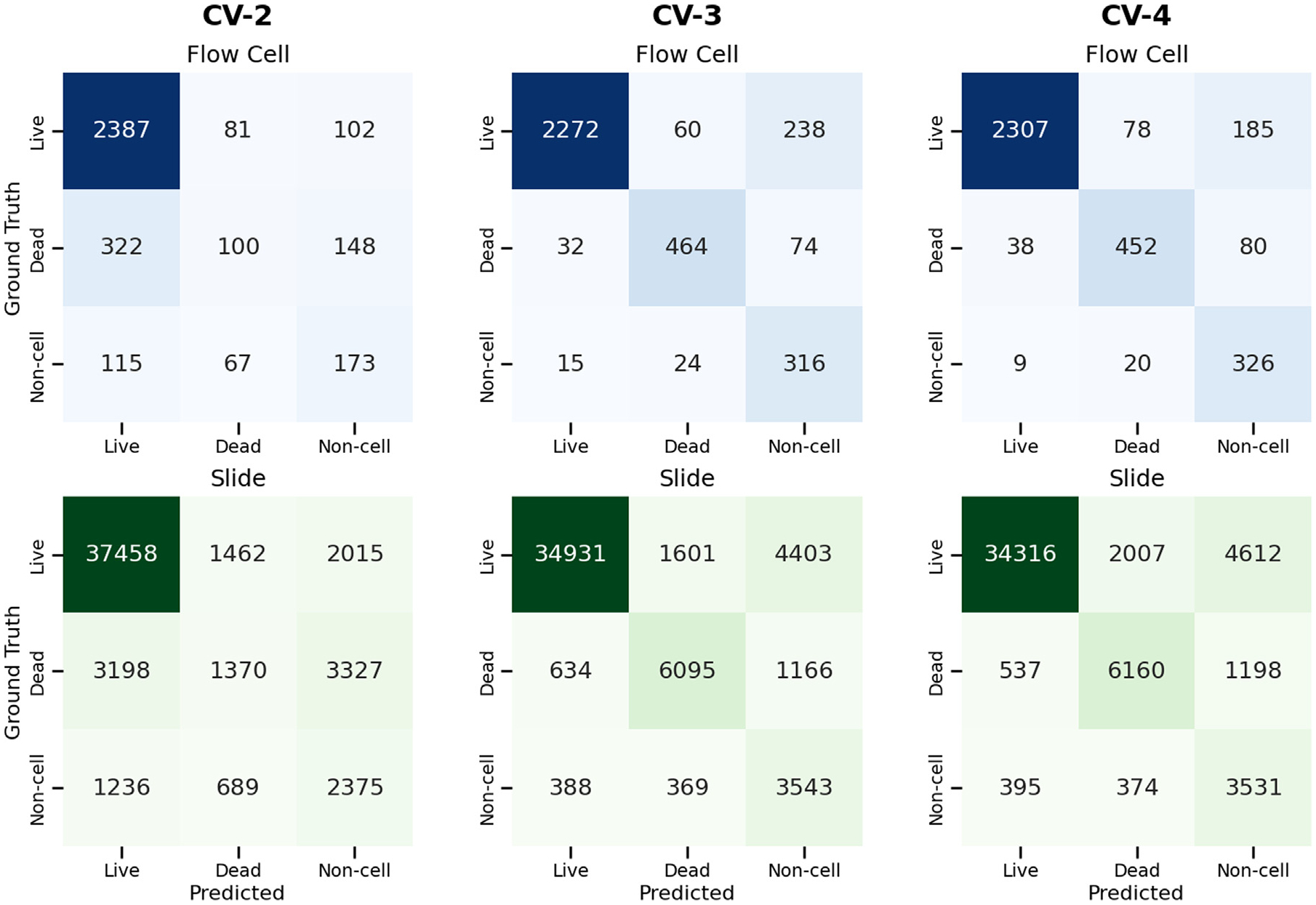
Internal validation object classification confusion matrices. Counts are aggregated across five repetitions. The top row represents the flow cell setting, and the bottom row represents the slide setting. The columns from left to right show CV-2, CV-3 and CV-4. Errors were more common with non-cellular object predictions, which inherently represent a higher-uncertainty class. CV-3 and CV-4 exhibit a substantial improvement over CV-2.

The internal validation performance for object-level classification is shown in Table 4 and the confusion matrix is shown in Figure 6. All prompts were highly effective at identifying live cells, with most of them achieving F1 scores above 0.9. In contrast, the classification of dead and non-cellular objects remained more challenging, with lower F1 scores across both conditions. Since object-level classifications are directly responsible for cell counting and viability estimation, the findings are mostly consistent with “Cell counting and viability estimation.” Overall, CV-3 and CV-4 provide improvements over CV-2 with substantially higher F1 scores for dead cells and non-cellular objects. The confusion matrix indicates that the inclusion of numerical features is important for the accurate classification of dead cells and non-cellular objects.

The classification results on the test dataset C are shown in Table 5. Compared to the development dataset, higher accuracy and F1 scores were obtained, likely reflecting the reduced complexity of the binary classification task. The accuracy of 0.94 achieved by CV-3 and CV-4 is comparable to the neural network baseline reported in Serafini *et al*. [18], which achieved an accuracy of 0.947 using a 70:30 train-test split.

**Fig. 7.**
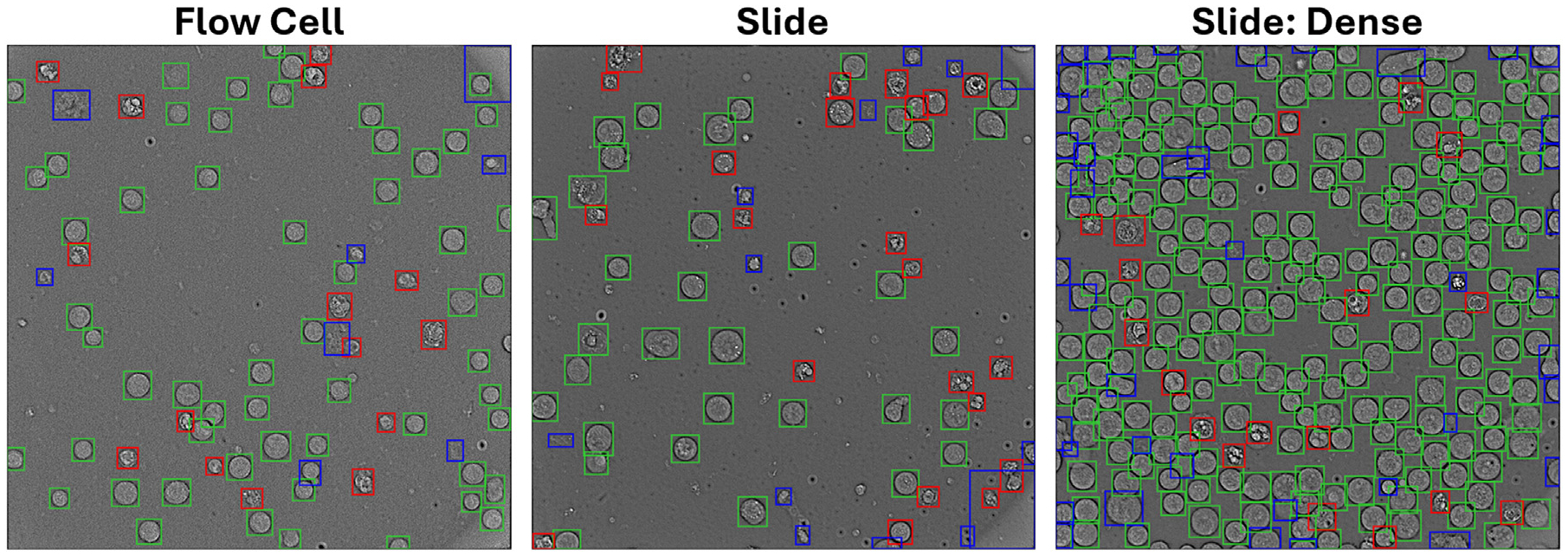
Example object classification results of CV-4 for live cells (green), dead cells (red) and non-cellular objects (blue) from the development dataset. The model classifies most objects correctly, but appears to assign more “non-cellular object” labels to objects on the edges of densely populated images.

Example classification predictions from the training dataset are shown in Figure 7. The model effectively captured the distinguishing features of different object types. Misclassifications typically occurred with objects that were more ambiguous or visually unclear. In a dense image with many objects, the model also tended to predict “non-cellular object” for objects on the edge of the image, possibly due to the corrupted morphological features.

**Fig. 8.**
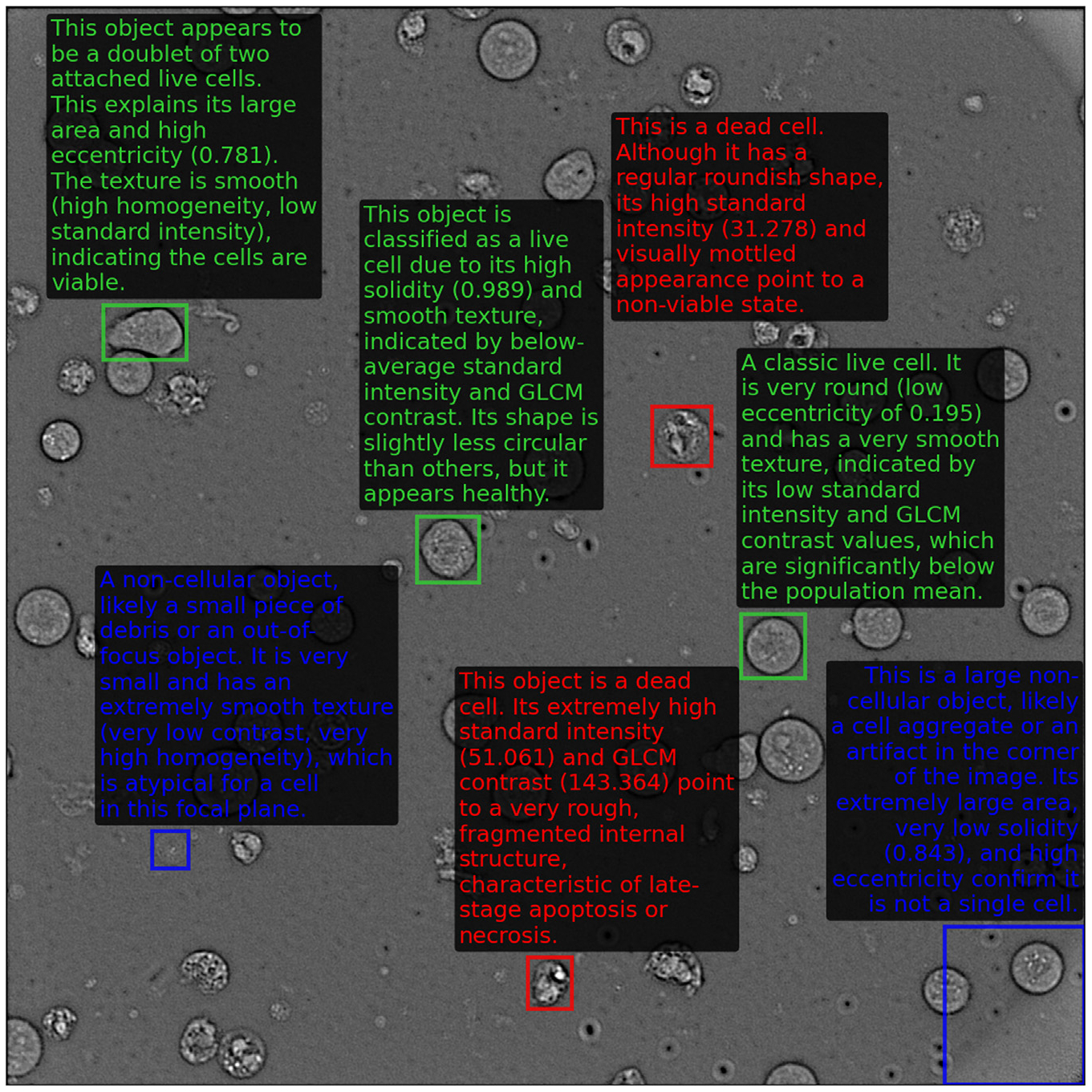
Example object classification rationales of CV-4 from the development dataset. The model examined both visual and numerical features, and used its existing domain knowledge to correctly classify a diverse set of objects within an image.

To make the classification process transparent, CV-4 required the model to provide a brief rationale for each object alongside its predicted label. Figure 8 illustrates examples of these explanations. In generating them, the model drew on both visual cues from the image and the numerical features supplied. Notably, even without explicit instructions on how to combine or weigh these features, the model was able to leverage its existing domain knowledge to justify its decisions in a way that is consistent with biological expectations.

### Cost analysis

At the time of experimentation, the paid tier of Gemini 2.5 Pro Application Programming Interface for short prompts (<200 000 tokens) costs $1.25 per million prompt tokens, and $10 per million thought and response tokens. Table 6 shows the costs of the different prompting strategies.

For anomaly detection, inference cost remained relatively consistent across the different prompt strategies. Interestingly, the most detailed prompt (AD-4) was actually the cheapest to run. This outcome is explained by its lower rate of false positive anomalies, which reduced the number of response tokens required. Furthermore, the additional guidance appeared to simplify the model’s reasoning, leading to a modest reduction in thought tokens as well.

For cell counting and viability prediction, the prompts consistently required more thought tokens than those used for anomaly detection. Introducing SAM objects led to a substantial increase in token use across all categories, as the model had to reason over a much larger input space. Adding numerical features further increased both prompt and thought token counts, reflecting the additional input data. However, the greatest cost came from the requirement for rationale generation in the output, which dramatically inflated the number of response tokens.

## Discussion

In this work, we introduced a zero-shot foundation model cascade for the automated analysis of microscopy images in cell therapy manufacturing, successfully performing anomaly detection, object detection, object classification, cell counting and viability estimation without any task-specific fine-tuning. Our results demonstrate that by strategically combining the strengths of a multimodal LLM and SAM, we can create a robust and interpretable system capable of handling the variability inherent in real-world manufacturing scenarios. The systematic ablation study further provides valuable insights into the effective design of prompting architectures for complex, domain-specific image analysis tasks.

A key advantage of this framework is its decoupled architecture for object localization and semantic reasoning. Traditional computer vision approaches often conflate these tasks within a single end-to-end model, such as U-Net or Mask R-CNN, requiring simultaneous learning of texture-based features for segmentation and morphological features for classification. By delegating segmentation to SAM, we leverage a model optimized for class-agnostic foreground–background separation, bypassing the limitations of current LLMs in dense, pixel-precise prediction. Conversely, using an LLM for classification overcomes the semantic limitations of SAM, which lacks biological knowledge of cell states. This modular design allows each foundation model to operate within its domain of expertise: SAM resolves structural ambiguity, while the LLM applies domain knowledge to interpret the extracted regions. Moreover, the cascaded structure supports modular upgrades, enabling individual components to be replaced as improved segmentation or reasoning models emerge.

A key finding of our work was the remarkable effectiveness of the LLM in performing zero-shot anomaly detection. Although a baseline prompt (AD-1) suffered from low precision due to an overeagerness to classify deviations as severe, incrementally adding context was highly effective. The best-performing prompt, AD-4, which included detailed descriptions of expected cell morphologies and potential contaminants, achieved a high accuracy of 0.95. This suggests that providing the LLM with explicit domain knowledge and clear definitions of severity allowed it to leverage its broad understanding to accurately identify true process anomalies, such as microbial contamination and excessive debris, while correctly dismissing minor deviations. This represents a significant advantage over traditional supervised learning models that would require extensive labeled examples of each anomaly type and often fail to generalize to novel anomalies. Furthermore, another major strength of this method is its inherent interpretability. Traditional methods often provide a score or classification without explaining the underlying reason. In contrast, our approach generates a detailed description of the anomaly, giving operators insight into the specific nature of a process deviation. In addition, the proposed method provides the flexibility to define which anomalies are severe enough to immediately alert the operator and which are minor, allowing analysis to continue uninterrupted.

In contrast to the straightforward success in anomaly detection, the tasks of cell counting and viability estimation presented a more nuanced challenge, revealing a delicate trade-off between holistic estimation and object-level precision. Initially, simpler prompts that instructed the LLM for direct counts (CV-1) yielded surprisingly competitive viability estimations, though they struggled with cell count accuracy. This indicates that although the LLM possesses a high-level understanding of cell population health from a gestalt view of the image, it is inherently poor at precise object enumeration, a known limitation of such models. The introduction of SAM for object-level grounding (CV-2) significantly improved cell counting accuracy by forcing the LLM to classify individual segmented objects, moving from an unreliable holistic estimation to a more structured, object-centric analysis. However, this initially worsened the performance of the viability estimation, perhaps partly due to the distraction of input bounding boxes and additional tasks in the prompt. The introduction of numerical features significantly improved object classification, leading to reduced errors in cell counting and viability estimation in the development dataset.

Providing the LLM with quantitative data such as object area, solidity, and texture features along with visual information allowed the model to achieve both low error in cell counting (<10% MAPE) and high accuracy in viability estimation (<0.05 MAE) in the development dataset. These results are encouraging, given that the FDA generally recommends a minimum viability of 0.7 for somatic cell therapies [66], and that validated analytical methods typically accept up to 10% coefficient of variation (around 14% MAPE) for cell counting accuracy [67] and absolute viability deviations of 0.10–0.15% [68]. This synergy highlights the advantage of using multimodal inputs. The LLM can integrate visual patterns with quantitative data to make more accurate classifications, outperforming approaches based only on visual features. This suggests that the LLM can effectively reason about the magnitudes of numerical values with its language-understanding capability. The final prompt (CV-4), which required the model to generate a rationale for each classification, not only maintained high performance but also allowed for more interpretable predictions. The model’s ability to produce biologically consistent justifications for its decisions, referencing both visual and numerical features, is a major step toward building trust and facilitating human-in-the-loop oversight in automated manufacturing systems.

The evaluation on test datasets A and B further supports the generalization capabilities of the proposed zero-shot cascade. Unlike supervised learning models, which may experience significant performance degradation when applied to data distributions differing from their training set, such as different donors or cell types, the foundation model cascade maintained competitive performance on therapeutically-relevant CAR T cells and isolated T-cell data without any exposure to these images during prompt development. Viability estimation errors were greater in the training set, likely reflecting differences between the flow cytometry–based ground truth and the image sampling strategy. This effect was exacerbated in dataset A by lower cell densities, which further increase sampling variability and reduce the contextual information available to the LLM. The latter interpretation is further supported by degraded performance in CV-3 and CV-4, which incorporate numerical features, as seen in Table 3 and Figure 5. These features are primarily informative in a relative sense, across multiple objects, and therefore become less effective when fewer objects are present. In this regime, they may also introduce additional input tokens that can negatively impact LLM performance. Nevertheless, errors below 10% were still achieved. This suggests that the foundation models can generalize to different cell types and experimental conditions.

Moreover, the results on dataset C demonstrate that the zero-shot approach can detect all labeled cells, and achieve binary classification accuracy comparable to fully supervised methods. On this dataset, the LLM achieved an accuracy of 0.94, effectively matching the performance of a task-specific neural network trained on 70% of the data [18]. This highlights that foundation model cascades can eliminate the need for the labor-intensive annotation and training cycles required by traditional machine learning.

Despite these promising results, this study is subject to several limitations. First, the datasets are relatively limited with restricted anomaly diversity. Specifically, only 22 images contained severe anomalies, which constrains the statistical power to rigorously assess performance on rare failure modes. Second, the experiments were confined to a single imaging modality and similar cell types. Consequently, the model’s robustness against realistic domain shifts, such as those arising from independent bioreactors, variations in imaging hardware, distinct cell products, or environmental perturbations, remains unverified. Future work would benefit from validation on larger, more heterogeneous datasets that capture this broader spectrum of manufacturing variability.

Furthermore, the classification of dead cells and non-cellular debris remained challenging across all prompting strategies, as reflected in their lower F1 scores compared with live cells. These objects often have ambiguous morphologies, making them difficult to classify even for human experts. The challenge is further exacerbated by limited contextual information: within each image, only a small number of dead cells or non-cellular objects are typically present, restricting the model’s ability to form relative or contrastive judgments for classification. This limitation is further highlighted by dataset A, where lower object counts per image contributed to higher errors in viability prediction, specifically in the per-image evaluation. In such low-context regimes, the model may not effectively calibrate class boundaries from sparse examples. Future work could address these limitations by incorporating richer feature representations or few-shot learning strategies, which explicitly leverage a few in-context examples to improve prediction accuracy.

Finally, the current cascade operates on a deterministic “halt-on-anomaly” logic without robust confidence scoring outside the LLM provided “severity,” Although this design acts as a fail-safe to prevent downstream models from generating meaningless metrics based on corrupted data, it necessitates a human-in-the-loop to verify alerts and manually override false positives to resume the cascade. The development of a comprehensive alarm management plan, including calibrated decision thresholds, uncertainty estimation, and protocols for operator escalation, is a critical requirement for operational deployment. Future work should explore integrating robust confidence outputs to support these decision-making processes and optimize the balance between sensitivity and operator workload.

Beyond analytical performance, the practical implementation of this cascade also requires careful consideration of computational cost and regulatory compliance. The cost analysis of the cell counting and viability prediction tasks reveals a clear trade-off between performance, interpretability, and expense. While the most accurate prompts (CV-3 and CV-4) offer superior results, the requirement in CV-4 to generate a textual rationale for each of the hundreds of objects in an image dramatically increases token consumption and inference cost. However, in a regulated manufacturing environment, the additional cost may be justified, and will become increasingly so as LLM inference costs continue to decrease. A significant barrier to adopting artificial intelligence in regulated manufacturing environments is the “black box” problem. The interpretability offered by CV-4 provides a human-readable audit trail for every classification, which could be invaluable for process validation, root cause analysis of deviations, and submissions to regulatory bodies. Therefore, although CV-3 presents a cost-effective solution for high-throughput screening, CV-4 represents a more suitable framework for deployment in a validated, quality-controlled production environment where transparency and traceability are important.

### Conclusions

In this work, we proposed a foundation model cascade for the zero-shot analysis of CQAs in cell therapy manufacturing. By systematically engineering a prompting strategy, we have shown that it is possible to guide a general-purpose LLM with vision capabilities to perform multi-faceted analysis of microscopy images, including anomaly detection, cell counting, and viability estimation, without any task-specific fine-tuning or data labeling. Our findings suggest that the key to unlocking the potential of these models lies not only in prompt engineering, but in a deliberate architectural design that decomposes complex tasks, leverages the LLM’s extensive pre-trained knowledge, integrates specialist segmentation foundation models, and enriches the LLM’s reasoning with multimodal inputs.

This zero-shot, interpretable approach presents a flexible and powerful alternative to traditional supervised learning methods, which are often brittle and require extensive effort to adapt to new conditions. As foundation models become even more powerful and their visual understanding and reasoning As capabilities continue to advance, the performance of a similar framework is poised to improve in tandem. This work marks a step toward more agile, automated, and intelligent process analytical technologies, paving the way for scalable, reliable cell therapy manufacturing.

## Supplementary Material

1

Supplementary material associated with this article can be found, in the online version, at doi:10.1016/j.jcyt.2026.102078.

## Figures and Tables

**Table 1:** Anomaly detection performance of the different prompting strategies.

Prompt	Accuracy	Precision	Recall	F1 score
AD-1	0.30 ± 0.02	0.21 ± 0.01	**0.98** ± 0.02	0.35 ± 0.01
AD-2	0.81 ± 0.02	0.50 ± 0.03	0.91 ± 0.00	0.64 ± 0.03
AD-3	0.94 ± 0.02	0.79 ± 0.05	0.90 ± 0.02	0.84 ± 0.04
AD-4	**0.95** ± 0.01	**0.82** ± 0.03	0.95 ± 0.02	**0.88** ± 0.02

The mean and standard deviation across five repetitions are reported. In each column, the bold font denotes the best value. Performance generally improves as the prompt evolves to be more comprehensive.

**Table 2:** Cell count mean absolute percent error MAPE and viability MAE from internal validation of the different prompting strategies.

Prompt	Flow Cell		Slide	
	Cell count MAPE (%)	Viability MAE	Cell count MAPE (%)	Viability MAE
CV-1	12.1 ± 1.5	0.038 ± 0.005	15.8 ± 0.7	0.054 ± 0.004
CV-2	13.1 ± 1.7	0.103 ± 0.009	11.5 ± 0.6	0.090 ± 0.002
CV-3	9.9 ± 1.3	0.035 ± 0.002	**9.5** ± 0.3	**0.041** ± 0.002
CV-4	**8.8** ± 1.7	**0.031** ± 0.006	9.8 ± 0.4	0.044 ± 0.001

The mean and standard deviation across five repetitions are reported. In each column, the bold font denotes the best value. The inclusion of numerical features (CV-3 and CV-4) substantially reduced errors.

**Table 3:** Zero-shot generalization viability MAE of the different prompting strategies on external datasets A and B.

Prompt	Dataset A: isolated culture		Dataset B: CAR T cells	
	Per-image	Per-sample	Per-image	Per-sample
CV-1	0.108 ± 0.003	0.094 ± 0.004	0.084 ± 0.002	0.078 ± 0.002
CV-2	**0.083** ± 0.002	**0.064** ± 0.004	0.109 ± 0.002	0.099 ± 0.003
CV-3	0.106 ± 0.002	0.090 ± 0.003	**0.081** ± 0.003	**0.075** ± 0.003
CV-4	0.105 ± 0.003	0.088 ± 0.004	0.089 ± 0.002	0.082 ± 0.002

The mean and standard deviation across five repetitions are reported. In each column, the bold font denotes the best value. Per-sample aggregation of images per-sample reduced errors.

**Table 4:** Object classification accuracy and F1 scores from internal validation of the SAM-based prompting strategies.

	Flow Cell				Slide			
Prompt	Accuracy	F1 score			Accuracy	F1 score		
		Live	Dead	Non-cell		Live	Dead	Non-cell
CV-2	0.76 ± 0.01	0.89 ± 0.01	0.24 ± 0.05	0.45 ± 0.03	0.78 ± 0.00	0.90 ± 0.00	0.24 ± 0.01	0.40 ± 0.00
CV-3	0.87 ± 0.01	0.93 ± 0.01	**0.83** ± 0.01	0.64 ± 0.02	**0.84** ± 0.00	**0.91** ± 0.00	**0.76** ± 0.01	**0.53** ± 0.00
CV-4	**0.88** ± 0.02	**0.94** ± 0.01	0.81 ± 0.01	**0.69** ± 0.04	0.83 ± 0.00	0.90 ± 0.00	0.75 ± 0.01	0.52 ± 0.01

The mean and standard deviation across five repetitions are reported. In each column, the bold font denotes the best value. The addition of numerical features in CV-3 and CV-4 led to substantial performance improvements. While accurately classifying non-cellular objects remained challenging, cell count error remained acceptable.

**Table 5:** Zero-shot generalization object classification accuracy and F1 scores on external dataset C.

Prompt	Accuracy	F1 score	
		Live	Dead
CV-2	0.88 ± 0.01	0.93 ± 0.00	0.45 ± 0.02
CV-3	0.94 ± 0.00	0.96 ± 0.00	0.80 ± 0.01
CV-4	**0.94** ± 0.01	**0.96** ± 0.00	**0.80** ± 0.02

In each column, the bold font denotes the best value. Consistent with the development dataset, the addition of numerical features in CV-3 and CV-4 led to substantial performance improvements.

**Table 6:** Comparison of token usage and Application Programming Interface costs for anomaly detection and cell counting/viability prediction on the development dataset.

Prompt	Prompt (K)	Thought(K)	Response (K)	Cost ($)
AD-1	0.36 ± 0.00	1.32 ± 0.01	0.16 ± 0.00	0.015 ± 0.000
AD-2	0.42 ± 0.00	1.31 ± 0.01	0.08 ± 0.00	0.014 ± 0.000
AD-3	0.46 ± 0.00	1.17 ± 0.02	0.05 ± 0.00	0.013 ± 0.000
AD-4	0.61 ± 0.00	1.21 ± 0.02	0.05 ± 0.00	0.013 ± 0.000
CV-1	0.33 ± 0.00	4.12 ± 0.10	0.03 ± 0.00	0.042 ± 0.001
CV-2	6.79 ± 0.00	7.54 ± 0.10	1.36 ± 0.00	0.097 ± 0.001
CV-3	19.09 ± 0.00	8.83 ± 0.13	1.36 ± 0.00	0.126 ± 0.001
CV-4	19.16 ± 0.00	9.58 ± 0.11	7.75 ± 0.07	0.197 ± 0.001

The mean and standard deviation of the average cost across five repetitions are reported.
