# Supplementary material for: Foundation model cascades enable zero-shot microscopy image analysis for cell therapy manufacturing
Source: Cytotherapy. Author manuscript; Available in PMC 2026 Jul 2. (PMC13325518; doi:10.1016/j.jcyt.2026.102078)
Supplement: 1 [file NIHMS2190668-supplement-1.pdf]

LLM Prompt System Instructions

AD-1

System Instructions: AD-1

Analyze the provided quantitative oblique back-illumination microscopy (qOBM) image of T-cells to detect anomalies, outputting a JSON list in the specified format where severity can be "Severe" or "Minor"; if no anomalies are found, output an empty list.

```
[
    {
      "Anomaly Type": "string",
      "Description": "string",
      "Severity": "string"
    }
]
```

AD-2

System Instructions: AD-2

Analyze the provided quantitative oblique back-illumination microscopy (qOBM) image of T-cells to detect anomalies, outputting a JSON list in the specified format where severity can be "Severe" or "Minor"; if no anomalies are found, output an empty list.

Expected image content includes primarily live and dead T-cells, with a small number of non-cellular objects (specks, bubbles) being normal. Severe cell death (>20% dead cells) should be labeled as a "Minor" anomaly unless other unexpected issues are present.

```
[
    {
      "Anomaly Type": "string",
      "Description": "string",
      "Severity": "string"
    }
]
```

AD-3

System Instructions: AD-3

Analyze the provided quantitative oblique back-illumination microscopy (qOBM) image of T-cells to detect anomalies, outputting a JSON list in the specified format where severity can be "Severe" or "Minor"; if no anomalies are found, output an empty list.

Expected image content includes primarily live and dead T-cells, with a small number of non-cellular objects (specks, bubbles) being normal.

Minor anomalies are slight deviations with minimal impact on analysis, such as excessive cell death (>20% dead cells). Severe anomalies are deviations that critically compromise the culture or prevent reliable analysis, such as Contamination, Pervasive Debris, Severe image artifacts, or any unforeseen anomaly critically impacting image integrity.

```
[
    {
      "Anomaly Type": "string",
      "Description": "string",
      "Severity": "string"
    }
]
```

AD-4

System Instructions: AD-4

Analyze the provided quantitative oblique back-illumination microscopy (qOBM) image of T-cells to detect anomalies, outputting a JSON list in the specified format where severity can be "Severe" or "Minor"; if no anomalies are found, output an empty list.

Expected image content includes primarily live and dead T-cells, with a small number of non-cellular objects (specks, bubbles) being normal. These non-cellular specks or bubbles are typically dust on the camera lens or mirror, appearing as very dark dots or short streaks encased in a brighter halo. Normal T-cells are usually spherical, but can occasionally be elongated or irregularly shaped.

Minor anomalies are slight deviations with minimal impact on analysis, such as excessive cell death (>20% dead cells). Severe anomalies are deviations that critically compromise the culture or prevent reliable analysis. Examples include Contamination (any visual evidence of foreign biological entities like bacteria as tiny motile dots, fungi as branched filaments or budding ovals, mycoplasma as diffuse extranuclear speckles, or morphologically distinct cross-contaminating cells); Pervasive Debris (significant presence of non-biological artifacts that obscure cells, such as dust, plastic, precipitated media, and air bubbles, typically appearing as sharply edged, refractile, or crystalline artifacts); Severe image artifacts (e.g., major focus issues, large scratches) that prevent analysis; or any unforeseen anomaly that critically impacts the image's integrity.

```
[
    {
      "Anomaly Type": "string",
      "Description": "string",
      "Severity": "string"
    }
]
```

CV-1

System Instructions: CV-1

Count the live and dead cells in the provided quantitative oblique back-illumination microscopy (qOBM) image of T-cells, outputting a JSON object in the specified format.

```
{
    "live_cell_count": number,
    "dead_cell_count": number
}
```

CV-2

System Instructions: CV-2

Classify the objects in the provided quantitative oblique back-illumination microscopy (qOBM) image of T-cells as "live cell," "dead cell," or "non-cellular object". You will be provided with an image and a list of detected objects, each with a unique identifier and a corresponding bounding box with coordinates normalized from 0 to 1000.

Your output MUST be a single JSON map, where the keys are the object `ids` and the values are the corresponding integer class labels: 0 for a live cell, 1 for a dead cell, and 2 for a non-cellular object. Every object 'id' from the input must appear as a key exactly once in the output map.

Input format:

- `id`: A unique identifier for the object.
- `box_2d`: An array of four numbers `[ymin, xmin, ymax, xmax]`.

Example output:

```
{
    "obj_0": 0,
    "obj_1": 0,
    "obj_2": 2,
    "obj_3": 1,
    ...
}
```

CV-3

System Instructions: CV-3

Classify the objects in the provided quantitative oblique back-illumination microscopy (qOBM) image of T-cells as "live cell," "dead cell," or "non-cellular object". You will be provided with an image and a list of detected objects, each with corresponding numerical features and a bounding box with coordinates normalized from 0 to 1000. You will also be provided with feature statistics describing the mean and standard deviation for each numerical feature across all detected objects.

Your output MUST be a single JSON map, where the keys are the object `ids` and the values are the corresponding integer class labels: 0 for a live cell, 1 for a dead cell, and 2 for a non-cellular object. Every object 'id' from the input must appear as a key exactly once in the output map.

Input format:
A JSON object for feature statistics.
A JSON list containing the objects to be classified, each with the following key:

- `id`: A unique identifier for the object.
- `box_2d`: An array of four numbers `[ymin, xmin, ymax, xmax]`.
- `objectness`: A score from 0.0 to 1.0 indicating detection confidence.
- `area`: The object's area in pixels.
- `solidity`: The ratio of the object's area to its convex hull area (0.0-1.0).
- `eccentricity`: A measure of how much the object deviates from being circular (0.0-1.0).
- `std_intensity`: The standard deviation of pixel intensities within the object.
- `glcm_contrast`: A measure of local intensity variations (texture).
- `glcm_homogeneity`: A measure of the uniformity of intensity (texture).

Example output:

```
{
    "obj_0": 0,
    "obj_1": 0,
    "obj_2": 2,
    "obj_3": 1,
    ...
}
```

CV-4

System Instructions: CV-4

Classify the objects in the provided quantitative oblique back-illumination microscopy (qOBM) image of T-cells as "live cell," "dead cell," or "non-cellular object". You will be provided with an image and a list of detected objects, each with corresponding numerical features and a bounding box with coordinates normalized from 0 to 1000. You will also be provided with feature statistics describing the mean and standard deviation for each numerical feature across all detected objects.

Your output MUST be a JSON list of objects, where each object contains three keys: `id`, `label`, and `desc`. Every object 'id' from the input must appear as a key exactly once in the output list.

- `id`: A unique identifier for the object.
- `label`: The corresponding integer class label: 0 for a live cell, 1 for a dead cell, and 2 for a non-cellular object.
- `desc`: A brief description of the rationale for the object's classification based on its numerical features and visual characteristics from the image.

Input format:
A JSON object for feature statistics.
A JSON list containing the objects to be classified, each with the following key:

- `id`: A unique identifier for the object.
- `box_2d`: An array of four numbers `[ymin, xmin, ymax, xmax]`.
- `objectness`: A score from 0.0 to 1.0 indicating detection confidence.
- `area`: The object's area in pixels.
- `solidity`: The ratio of the object's area to its convex hull area (0.0-1.0).
- `eccentricity`: A measure of how much the object deviates from being circular (0.0-1.0).
- `std_intensity`: The standard deviation of pixel intensities within the object.
- `glcm_contrast`: A measure of local intensity variations (texture).
- `glcm_homogeneity`: A measure of the uniformity of intensity (texture).

Example output:

```
[
    {
        "id": "obj_0",
        "label": 0,
        "desc": "Appears as a round object with a smooth texture. High solidity
                 and low eccentricity are consistent with the visual morphology
                 of a healthy, live cell."
    },
    ...
]
```

Impact of Domain Knowledge Guidance in Cell Counting and Viability Estimation

We also evaluated a version of the CV prompt to test whether including domain knowledge as guidance could improve performance. Building on CV-1, this version added descriptions of live, dead, and non-cellular objects to try to help the LLM differentiate cell states. Live cells are smooth or mildly textured with a rounded shape, while dead cells have speckled interiors and broken halos. Non-cellular objects are small, blurry, or irregular. Counts are still output as a simple JavaScript Object Notation (JSON) object. This prompt version is referred to as CV-1A.

CV-1A System Instructions

System Instructions: CV-1A

Count the live and dead cells in the provided quantitative oblique back-illumination microscopy (qOBM) image of T-cells, outputting a JSON object in the specified format.

Cells are distinct, cohesive objects, while non-cellular ones are very small, blurry, fragmented, clustered, or irregular artifacts. Live T-cells have smooth or mildly textured interiors with a continuous halo and rounded shape. Dead T-cells show shattered or speckled interiors with sharp contrast, often with a broken halo or irregular outline.

```
{
    "live_cell_count": number,
    "dead_cell_count": number
}
```

CV-1A Results

As seen in Table 1A, the inclusion of domain knowledge guidance worsened performance. This was likely because the visual diversity of the dataset, which spanned several imaging conditions and their associated batch effects, rendered a single set of descriptive rules for object classification insufficient and potentially restrictive. The model was more effective when allowed to leverage its own pre-trained knowledge on the full image context rather than being constrained by narrow definitions. For this reason, the CV prompts did not include domain knowledge and relied mostly on the model's existing knowledge for classification.

Table 1A

Cell count Mean Absolute Percent Error (MAPE) and viability Mean Absolute Error (MAE) of CV-1 compared to CV-1A in the development dataset.

Prompt	Flow Cell		Slide	
	Cell Count MAPE (%)	Viability MAE	Cell Count MAPE (%)	Viability MAE
CV-1	12.1 ± 1.5	0.038 ± 0.005	15.8 ± 0.7	0.054 ± 0.004
CV-1A	19.3 ± 4.2	0.044 ± 0.010	24.8 ± 0.6	0.054 ± 0.002

The mean and standard deviation across five repetitions are reported.
